# Biocompatible Thin Films Deposited by Laser Techniques

**DOI:** 10.3390/ma19050925

**Published:** 2026-02-28

**Authors:** Andrei Teodor Matei, Anita Ioana Visan

**Affiliations:** 1 IT Center for Science and Technology, 011702 Bucharest, Romania; matei.andrei5@gmail.com; 2 National Institute for Lasers, Plasma and Radiation Physics, 077125 Măgurele, Romania

**Keywords:** biocompatible thin films, laser deposition, surface engineering, film stoichiometry, implantable devices

## Abstract

Biocompatible thin films are essential for advancing biomedical devices, as they enhance integration with biological tissues, improve device longevity, and reduce complications. The rapid evolution of both medical needs and materials science has led to a diverse array of deposition techniques, each offering unique advantages and challenges for tailoring surface properties without compromising the bulk characteristics of implants and sensors. While laser-based methods—such as pulsed laser deposition (PLD) and Matrix-Assisted Pulsed Laser Evaporation (MAPLE)—are renowned for their precision, ability to preserve complex material stoichiometry, and suitability for low-temperature processing, the broader landscape includes several other important approaches. Physical Vapor Deposition (PVD) techniques, including magnetron sputtering and pulsed electron deposition, are widely used for their ability to create uniform, adherent coatings with controlled thickness and composition, making them suitable for both hard and soft biomedical substrates. Chemical Vapor Deposition (CVD) and its plasma-enhanced variant (PECVD) offer conformal coatings and excellent control over film chemistry, which is particularly valuable for functional polymer and ceramic films. Other methods, such as sol–gel processing, ion beam deposition, and electrophoretic deposition, provide additional flexibility in terms of coating composition, adhesion, and processing temperature, allowing for the fabrication of films with tailored mechanical, chemical, and biological properties. Despite these advances, the field faces ongoing challenges in optimizing film properties for specific clinical applications, ensuring reproducibility, and scaling up production for widespread use. The necessity of this review lies in its comprehensive comparison of laser-based techniques with alternative deposition methods, providing critical insights into their respective strengths, limitations, and suitability for different biomedical scenarios. By synthesizing recent developments and highlighting current gaps, this review aims to guide researchers and clinicians in selecting the most appropriate thin-film deposition strategies to meet the evolving demands of next-generation biomedical devices.

## 1. Introduction

A thin film represents a thin layer of a material that is generally used to enhance the surface properties of solids [1,2]. Coating is the procedure that incorporates a thin film of material into a substrate using deposition techniques [3]. The structure of deposited thin films can be broadly divided into amorphous and crystalline [1]. Each of these types of thin films can improve the surface of a bulk surface by tailoring a significant number of properties, including corrosion resistance, transmission, abrasion resistance, electrical behavior, biocompatibility, hardness, and so on [2,4]. In this way, thin films are widely used in several applications such as electrical devices [5], membranes [6], chromatography [7], electrochemistry [8], separation [9], surface engineering [10], drug delivery [11], tissue engineering [12], advanced coatings for medical purposes [13], and so on.

In the biomedical context, the surface of a medical device or implant is crucial for determining a biological response as it interacts with cells and proteins [14,15,16]. In this way, thin film coatings are widely used for their immediate biocompatibility and colloidal stability, which promote tissue integration, reduce corrosion, and limit bacterial adhesion and biofilm formation [16,17]. Depending on the specific application, different types of thin films, including metallic, ceramic, polymeric, or hybrid coatings, are employed to enhance mechanical and biological properties and performance in order to achieve the clinical requirements for precisely controlled deposition strategies [4].

Moreover, thin film coatings can decouple bulk and surface properties: metallic or ceramic substrates provide mechanical strength, while tailored surface layers control corrosion, ion release, cell adhesion, and microbial colonization [4]. For metallic biomaterials such as AISI 316L stainless steel or Ti alloys, thin films mitigate corrosion and toxic ion release (e.g., Ni, Cr) while tuning roughness and wettability to promote osseointegration [18]. Hybrid sol–gel/sputtered Ti films on 316L stainless steel, for example, significantly reduce roughness and adjust the contact angle into the moderately hydrophilic range, supporting stem cell adhesion and showing no cytotoxicity over 14 days [19]. Coatings can simultaneously impart bioactivity (e.g., apatite-forming calcium phosphates, bioactive glasses), antimicrobial effects (e.g., Ag, Cu, antibiotics, natural compounds), and corrosion protection, which is increasingly viewed as a prerequisite for long-term clinical success [20]. Multifunctional architectures—such as bioglass/antibiotic/polymer stacks or drug-loaded polymer matrices on nanotubular Ti—achieve sustained release while maintaining cell viability and improving electrochemical stability in simulated body fluid [21].

Current deposition techniques are mainly divided into Chemical Vapor Deposition (CVD) and physical vapor deposition (PVD) [2]. However, a wide range of other approaches, such as dip-coating, spin-coating, or electrophoretic deposition, are employed, in particular for bioactive materials [4,22]. While these methods offer scalability and cost-effectiveness, they are limited in several important aspects, such as stoichiometric precision control or compatibility with complex materials [23]. In this context, laser-based deposition techniques such as pulsed laser deposition (PLD) or Matrix-Assisted Pulsed Laser Evaporation (MAPLE) represent suitable alternatives for developing thin films with precise control over thickness, composition, and microstructure under highly controlled conditions [24,25].

This review focuses on how different classes of thin films, such as metallic, ceramic, polymeric, or hybrid, are optimally processed by specific deposition techniques, with particular emphasis on laser-based deposition approaches such as PLD and MAPLE, highlighting the most optimal strategies for recent and emerging biomedical applications of advanced thin film coatings reported in the literature. The central premise of this review is that the choice of a deposition technique is not merely a fabrication decision but a fundamental design step that dictates the eventual biological performance of a medical device. Rather than presenting these methods as parallel fabrication routes, we frame them as strategic design tools for engineering specific surface functionalities—such as controlled drug release, enhanced osseointegration, or infection resistance—by directly linking process parameters to the desired clinical outcome. This review will therefore examine how the unique capabilities of each deposition class (PVD, CVD, solution-based, and laser-based) can be strategically leveraged to process distinct material classes (metallic, ceramic, and polymeric) and achieve targeted biological and mechanical performance.

## 2. Overview of Thin Film Deposition Techniques for Biomedical Applications

Thin film deposition techniques describe a process of coating surfaces of a substitute material with layers that range in thickness from a few nanometers to 1 μm [26,27]. These deposition techniques are fundamental for determining several materials with unique properties, which find their applications in several domains such as chemistry, engineering, research, and physics [28]. These unique properties and the technological potential of materials fabricated as thin films have gained interest throughout the years. Thus, there are a significant number of fabrication methods that have been analyzed and tested to determine the most suitable and efficient method [26].

**Figure 1:**
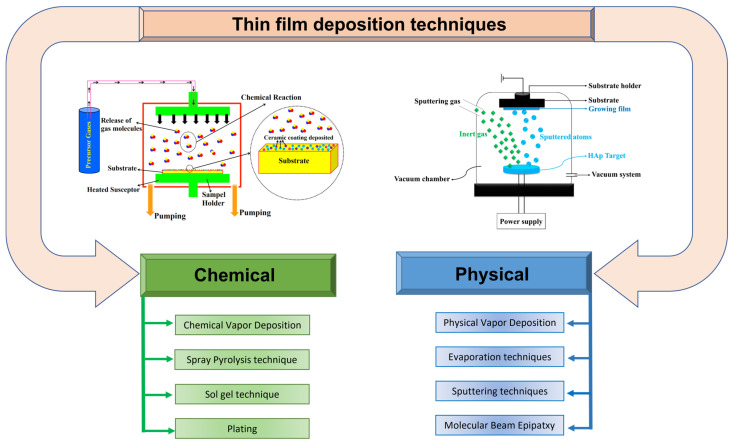
Overview of chemical and physical thin film deposition techniques, including representative deposition mechanisms. Chemical methods involve precursor decomposition and chemical reactions at the substrate surface (e.g., CVD, spray pyrolysis, sol–gel, and plating), while physical methods rely on vaporization and condensation of material species under vacuum conditions (e.g., PVD, evaporation, sputtering, and molecular beam epitaxy). Reproduced from [30,31].

In this way, choosing the right deposition method depends on the material, the device, and the clinical need. To understand how these choices translate into clinical function, this section provides an overview of the main deposition categories. Each subsection will not only describe the fundamental mechanism of a technique but will also frame it as a strategic tool, highlighting how its specific process parameters can be tuned to engineer surface properties relevant for biomedical applications. Depending on the mechanism by which the material is deposited, techniques fall into two main categories: physical deposition techniques and chemical deposition techniques [29]. The most common physical and chemical deposition techniques are represented in Figure 1.

### 2.1. Physical Vapor Deposition (PVD)

Physical Vapor Deposition (PVD) represents a deposition technique that is based on the vaporization of a material to the state of atoms or molecules and on the further transport in a state of vapor to a substrate [32]. The fundamentals of this process consist of setting up the environmental conditions to ensure precise control over several key parameters such as pressure and temperature [33]. This technique is usually used to deposit thin films with thickness in the range of a few micrometers to a few nanometers [32,34]. It relies on distinct physical mechanisms to create the vaporized species following the fundamental concept of transportation and condensation onto the substrate. Coatings produced by PVD are commonly characterized by long-term stability and strong adhesion, determining significantly improved properties such as enhanced corrosion resistance, increased hardness, or modified surface appearance [33]. PVD encompasses sputtering, cathodic arc, pulsed electron deposition, PLD, and related methods that eject atoms or clusters from a solid target and condense them on the implant surface [35]. Recent progress in PVD technology suggests the development of nanostructured materials and advanced deposition techniques, and these innovations have determined a wide expansion of the applications of PVD coatings, including surface refinement of 3D printed components, renewable energy systems, or electric vehicle components. The future of PVD tends to be prioritized on the fabrication of sustainable and eco-friendly materials, and considering the implementation of in situ monitoring systems, this will ensure precise controlled coating determining several complex geometries [33].

In the context of biomedical coatings, PVD is recognized as a safe and efficient technique that has been used to produce biocompatible coatings that improve the quality of a biomaterial or a medical device by strengthening the corrosion resistance and their performance [36]. Biomaterials require several characteristics in order to be successfully employed in medical applications, and coating the surface with PVD could reduce wear and friction and enhance biocompatibility and osteointegration [37]. Medical devices such as surgical instruments, implantable devices, or interventional devices are suitable applications for coating with PVD, improving their performance and extending their lifetime [38]. Also, in biomedical coatings, radio-frequency magnetron sputtering and sputter-variants are widely used due to their ability to deposit dense, adherent films with well-controlled microstructure on temperature-sensitive metallic substrates [39]. Sputtering of Ti_3_Au intermetallics doped with Ag or Cu, for example, produces super-hard coatings (a specific hardness of 14–15 GPa; ~3–3.5× Ti_6_Al_4_V) with excellent corrosion resistance and rapid bactericidal activity at ≤0.5 at% dopant while retaining low cytotoxic ion release, as measured by nanoindentation [19]. Electron beam physical vapor deposition is an effective surface modification technique that enables deposition of adherent and uniform coatings on pure Mg without any contaminations under high vacuum and low temperature conditions, obtaining specific coating thickness to achieve enhanced surface smoothness [40].

PVD methods allow straightforward alloying and co-sputtering of multiple targets, enabling gradient or composite coatings and incorporation of bioactive ceramics or therapeutic agents into thin layers [41]. The advantages of this technique are unique and numerous; it implies superior functionality, controlled morphology, and the ability to develop structures with monolayers and multilayers. The drawback of PVD is mainly related to the substrate surface, which has to be easily accessible. Also, high capital cost and vacuum infrastructure remain barriers to large-scale deployment, even as standardized PVD systems become more accessible to industry [39,42].

### 2.2. Chemical Vapor Deposition (CVD)

Chemical Vapor Deposition (CVD) is a material processing technique in which a solid film structure is deposited on a substrate through chemical reactions of gaseous precursors on a heated surface or near it [43]. During this process, the precursor species are delivered to the substrate in gaseous form, where the precursor, usually, originates from a volatile liquid or, in rare cases, from a solid that is vaporized or sublimed [44,45]. This vapor is conveyed to the deposition area by an inert or reactive carrier gas [44]. CVD and its related techniques are mainly used to produce thin films and advanced materials that range from electronic and protective coatings to high-temperature materials [45], solar cells [46], and carbon nanotubes [47]. This technique allows the production of a significant number of metal or nonmetallic elements, and it is used in the production of fibers, powders, and coatings [48]. Thin films fabricated by CVD are characterized by high-purity deposits, improved adhesion, controlled roughness, corrosion resistance, friction reduction, erosion resistance, and heat resistance [43,48,49]. In recent years, CVD has been recognized as a valuable process for producing materials with high-quality properties in several domains, including microelectronics, semiconductor lasers, optical fibers, coatings with enhanced properties, and so on [43,45]. As a result, CVD technology is remarkable for providing superior conformal coverage and high-purity material layers on complex geometries, allowing precise control of deposition [43]. However, CVD presents some important drawbacks, such as chemical and safety hazards that usually occur during the process, the increased difficulty to deposit multicomponent materials with controlled stoichiometries, or the overall high cost [48].

In the biomedical domain, CVD is considered an essential tool for advancing the performance of medical implants, determining precise surface engineering. In this way, CVD provides layers with enhanced biocompatibility, mechanical strength, and overall functionality and performance [50]. Also, the performance of medical devices is improved by coating with CVD, especially with carbon-based materials [51]. This technology is critical in specialized fields where the safety and efficiency of an implant are prioritized, and sophisticated coatings are required for reducing the risk of clinical complications, extending the lifespan [50]. CVD and related vapor-phase chemistries (including ALD and molecular layer deposition, MLD) offer conformal, compositionally controlled coatings on complex, high-aspect-ratio geometries [50]. CVD-derived ceramic, carbon-based, or polymer-like films can enhance wear resistance, friction, and biocompatibility of load-bearing implants, with process parameter tuning (precursor chemistry, temperature, plasma conditions) enabling precise control of film density, bonding state, and residual stress [22]. Also, CVD can be beneficial in biosensing or microfluidic applications by parylene deposition [52].

ALD/MLD hybrids have been used to grow silicon-based hybrid alumosilazane nano-layers that exhibit excellent conformality on complex morphologies and support significantly higher proliferation of HEK293T cells than glass controls, demonstrating both mechanical flexibility and robust biocompatibility [22]. As a physical functionalization method, CVD is capable of generating a solid film with both homogenous and hierarchical structures [53]. Gas-phase MOF deposition (CVD or ALD/MLD routes) is emerging as a route to porous, drug-releasing, and corrosion-resistant films on metallic substrates, though long-term stability and controlled degradation remain active challenges [22].

### 2.3. Solution-Based and Other Methods

Sol–gel [54], dip-coating [55], electrophoretic deposition (EPD) [56], and related wet methods remain attractive due to low equipment cost and scalability [22,50]. The sol–gel technique serves as a versatile platform for synthesizing a wide range of functional oxide films [57]. It is suitable for depositing thin films on different types of materials, including metals and ceramics. Protective coatings have been deposited by the sol–gel technique on metals and alloy surfaces, which improved the corrosion resistance while being an environmentally friendly coating providing high oxidation and abrasion [58]. Regarding ceramics, sol–gel can be obtained from a variety of ceramics such as chromium, alumina, silica, zirconium, and so on. It has been determined that sol–gel coating films on ceramics enhanced the corrosion resistance and the microstructure of the material’s surface, leading to implementation in electronics and biomedical applications [59]. Sol–gel routes can produce bioactive, calcium-phosphate-based films at relatively low temperatures, though post-deposition calcination is often required to densify porous layers and improve adhesion [50]. This technique is used due to its cost-effectiveness and the ability to synthesize high-purity and adherent coatings at a relatively low temperature (200–600 °C). On the other hand, despite its versatility, this method is hindered by some limitations, such as limited coating thickness and lengthy durations required for drying and sintering phases [60].

Dip-coating represents an economical technique that consists of dipping a substrate into a liquid and removing it in a controlled manner [61,62]. This technique is widely used in various domains to deposit onto any substrate, such as metallic, polymer films, ceramic, etc. [61]. Metallic thin films fabricated by dip-coating offer a controlled, uniform thickness and consistent film properties [63]. For example, transparent and hydrophobic ZnO thin films can be deposited on glass substrates by dip-coating with low energy and reagent consumption [64]. Thin films can be obtained by dip-coating water-soluble polymers, obtaining coatings with controlled and uniform thickness and tunable optical and wettability properties [62]. Thus, dip-coating has the advantages of being a low-cost and versatile method, allowing facile applications of compounds, making it scalable [64,65]. The main disadvantage of dip-coating is related to the roughness of the surface, which is hard to control and could potentially affect variation in electrical resistance [65].

Electrophoretic deposition (EPD) represents a colloidal processing technique that is based on the electrophoretic motion of charged particles dispersed in a liquid medium. The fundamentals of this method rely on the migration of the particles when an external electric field is applied and the deposition of these particles in a controlled manner, leading to the deposition of thick films and coatings [66]. In this way, EPD has found the technique has found broad application in advanced ceramic processing and coating fabrication, as well as in the controlled manipulation and assembly of biological entities such as proteins, enzymes, and cells, across fields such as materials science, biotechnology, pharmaceutical sciences, chemistry, and biochemistry [66,67]. Coatings fabricated by EPD are characterized by uniform and controllable thickness, high packing density with low porosity, good adhesion to complex-shaped substrates, and a well-controlled microstructure [56,67]. Also, EPD enables rapid deposition of nanoparticle or polymer suspensions into uniform films over complex geometries but may demand high-temperature sintering and careful crack control [50]. Regarding this technique, the major advantage of electrophoretic deposition is its ability to produce uniform coatings using simple and low-cost equipment, while a key limitation consists of the necessity for carefully stabilized suspensions and electrically conductive substrates, which can restrict material selection and process robustness [67].

Hybrid schemes—e.g., sol–gel or silane-based layers followed by PVD Ti or ceramic overcoats—can exploit the strong chemical bonding of wet chemistries and the dense, controllable top layers afforded by vacuum techniques, leading to improved cell integration on stainless steel and Ti substrates [50]. Another example employs a model of current versus time for a hybrid scheme of sol–gel and electrophoretic deposition, which can offer a predictive model for precise regulation of deposition rates and film thickness, facilitating uniform and durable hybrid coatings [68].

### 2.4. Laser-Based Deposition Methods: Capabilities and Biomedical Relevance

Laser-based deposition methods are versatile techniques that offer a valuable tool for the fabrication of micro/nanostructures and advanced thin film deposition [69,70]. Their capability to preserve chemical integrity, enhance microstructure, and deposit complex inorganic, organic, or hybrid materials has attracted significant interest for biomedical applications such as coatings for implants, drug delivery layers, and bioactive surfaces [15]. Among these techniques, the most widely used and investigated techniques are pulsed laser deposition, Matrix-Assisted Pulsed Laser Evaporation, and other laser-enabled strategies, which will be further discussed.

#### 2.4.1. Pulsed Laser Deposition

PLD represents a thin-film fabrication method in which high-energy laser pulses are used to ablate a material from a solid target, creating a flux of ejected species that condense onto a substrate [25]. This process of deposition is carried out under vacuum conditions to limit scattering of the ablated species [25]. In PLD, high-energy laser pulses ablate a target, generating a plasma plume that deposits onto a substrate. The intrinsic non-equilibrium nature of PLD and the short interaction time facilitate near-stoichiometric transfer of complex materials—including multi-component oxides, intermetallics, and doped ceramics—often at relatively low substrate temperatures compared with conventional CVD [35]. Short laser pulses used to generate the depositing flux lead to distinctive features that make PLD suitable for vapor-phase growth processes [71]. In comparison with other plasma-assisted techniques such as plasma-enhanced CVD or plasma spraying, PLD represents a relatively simple, fast, flexible, and low-cost technique for producing high-quality coatings with enhanced performance from a variety of materials [25,71]. A typical PLD setup consists of a vacuum chamber equipped with at least one optical window transparent to the laser wavelength. In the interior of the chamber, the target and the substrate holder are positioned at a fixed distance where the target surface is oriented toward the substrate. When the laser is irradiating the target, the ablation produces a plasma plume composed of energetic species that expands toward the substrate [72]. In this way, PLD can yield dense, crystalline, or amorphous films depending on energy density and background gas pressure, with thicknesses from tens of nanometers to a few micrometers [73]. Coatings produced by PLD are characterized by dense, uniform, and homogenous structures that provide high continuity and superior bonding strength. These properties refine microstructures, determining mechanical properties and offering enhanced hardness and improved resistance to corrosion [74,75]. Considering this and despite the limitation of coatings large surfaces areas, PLD technique provide high interest due to its key advantages such as near-stoichiometric transfer of the target composition to the deposited film for bioactive coatings such as hydroxyapatite or doped bioceramics, where compositional fidelity directly influences osseointegration and corrosion resistance, strong adhesion between the coating, improved morphological uniformity, precise control over phase composition and thickness, reduced porosity along with lower propensity for cracking [25,76].

In biomedical contexts, lasers, in general, can provide a clean and fast process for a biomaterial or medical device. The fabrication of high-quality coatings over the surface of medical devices requires some critical characteristics, including the design and control of crystallinity, surface topology, and mechanical properties, which involve good adhesion, high hardness, and hard toughness [77]. Coatings fabricated with PLD are capable of covering these requirements due to their significant advantages and characteristics. PLD has been used to grow dense bioactive glass or calcium-phosphate coatings, titanium-based hard coatings, and polymeric or hybrid layers where thermal budget is limited [41]. Also, PLD is used for developing bioceramic thin films, which are widely applied in medical prostheses to tailor implant surface topography and biological performance [15]. Beyond bioceramic coatings, PLD can also be employed to fabricate thin polymer films. It has used to deposit hydroxyapatite films onto laser-sintered polyamide substrates, where precise adjustment of processing PLD parameters allows retention of polymer chemical functionalities and the formation of homogeneous layers with controlled thickness and surface features, leading to enhanced wettability and improved biocompatibility [78]. PLD also enables the fabrication of metal-containing thin films. For example, by the PLD technique, the deposition of Au-doped carbonated hydroxyapatite on alumina scaffolds has been developed, where controlled gold incorporation markedly altered the coating microstructure, surface roughness, and consequently cell adhesion and proliferation, highlighting the strong influence of compositional tuning on biological response [79]. In the same context, tantalum nitride films with enhanced performance related to crystallinity, mechanical properties, and corrosion can be precisely synthesized by PLD, developing high-quality coatings on dental or prosthetic implants [80]. PLD coatings are generally characterized by high adhesion and tunable roughness, both of which affect protein adsorption and early cell responses and are essential for long-term implant stability. However, elevated local temperatures and energetic species can complicate the direct incorporation of thermally labile biomolecules or drugs, imposing a trade-off between crystallinity and biofunctionalization [50]. This precise control over composition and microstructure, combined with the ability to deposit dense, adherent films from a wide variety of target materials, establishes PLD as a powerful technique for engineering stable, multifunctional, and biologically active surfaces that directly support the improved long-term performance of medical implants. Overall, coatings fabricated by PLD offer precise control over composition and microstructure, enabling the fabrication of stable, multifunctional, and biologically active surfaces that support improved long-term performance of medical implants. In this way, biocompatible coatings with enhanced performance can be achieved using PLD.

#### 2.4.2. Matrix-Assisted Pulsed Laser Evaporation

MAPLE is a laser-based PVD process that is mainly used for the fabrication of thin films with enhanced properties such as roughness, thickness, or homogeneity [81]. In this process, a pulsed laser beam irradiates a frozen target consisting of a dilute solution of the material to be deposited dissolved in a volatile solvent [82]. During laser irradiation, the solvent matrix preferentially absorbs the laser energy, encountering desorption without depositing on the substrate due to its low sticking coefficient [81]. MAPLE modifies PLD for soft matter: the solute (polymer, biomolecule, nanoparticle) is dissolved in a volatile solvent, frozen, and irradiated; the laser energy is largely absorbed by the matrix, desorbing intact macromolecules into the vapor phase [83]. The main significant difference between MAPLE and PLD is the absorption of the solvent by the laser beam energy while the molecules are ejected from the target, allowing the deposition of uniform and well-oriented films with precise control of thickness [82]. In MAPLE, the target used is a frozen dilute solution, setting up a softer mechanism with respect to PLD [82]. This “gentle” ablation regime has been extensively developed for organic, biological, and composite films, including proteins, enzymes, hydrogels, antibiotics, and functional polymers [41]. Considering this, the MAPLE deposition setup is similar to that used in PLD, incorporating excimer lasers or Nd:YAG operating lasers. The key difference lies in the target assembly, which must be maintained at cryogenic temperatures [84]. Thin films deposited by MAPLE present key characteristics such as surface morphology, tunability, high surface area, good adherence over large surfaces, material-dependent morphology, and ultra-thinness (from a few tens of nm up to 1 μm) [81,85,86]. Thus, MAPLE offers key advantages over traditional thin film deposition methods, including precise control over thickness, roughness, solvent-free deposition, the ability to pattern organic thin films, compatibility with scalable processing, and gentle deposition conditions that preserve the integrity of biological materials [81]. A recurring limitation is scalability: MAPLE is currently largely confined to laboratory and pilot-scale systems, with limited deposition areas and relatively modest growth rates. Nonetheless, advances in hybrid deposition systems, beam scanning, and combinatorial MAPLE approaches indicate potential for industrial translation in high-value biomedical devices where precise composition and biomolecule preservation are critical [41]. Also, rough films deposited by MAPLE, whose morphology depends on processing conditions and the intrinsic molecular structures, can be difficult to control precisely, especially in the case of biomaterials [86].

Regarding the biomedical domain, the MAPLE technique has been successfully used for the deposition of thin films of organic molecules, biopolymers, or composites [86]. Also, MAPLE could play a potentially significant role in enabling integration between metallic alloy substrates and biomimetic coatings, offering precise overall control over coatings. The development of a high-performance multifunctional biomimetic layer can be achieved by exploiting the complementary properties of different constituent materials [87]. For example, the deposition of an adherent biomimetic nanocrystal apatite thin film by MAPLE onto titanium and silicon substrates has been developed, demonstrating that the MAPLE technique is capable of securing the structural integrity of biomimetic apatite during its transfer from target to substrate [88]. Thin films of biocompatible polymers such as poly (D, L-lactide) (PDLLA) [89], PEG/PLLA [90], or PLA [91] have been successfully deposited using the MAPLE technique to be employed in different areas of medical domains, including osteosynthesis, medical implants, and tissue engineering [92]. Recent work shows MAPLE can deposit polymeric composite thin films loaded with metal nanoparticles (Ag, Au), metal oxides (TiO_2_, ZnO), antibiotics, or natural compounds (e.g., eugenol, isoflavonoids), achieving broad-spectrum antimicrobial activity via ion release, ROS generation, and membrane disruption while preserving the organic matrix [83]. Composite coatings developed by MAPLE involving hydroxyapatite and chitosan have proven enhanced cell adhesion and proliferation, sustaining the differentiation of pre-osteoblasts towards mature bone cells [92]. These coatings target implant-associated infections and antibiotic resistance, often combining bactericidal action with osteoconductive ceramics such as magnesium phosphate [93]. Multilayer MAPLE architectures—e.g., polymeric base layer followed by bioglass/antibiotic top layers—on Ti implants yield coatings that are both biocompatible and highly resistant to microbial colonization and biofilm formation in vitro [20]. Similar MAPLE-deposited bioglass–melittin composite films on Ti convert hydrophobic surfaces to moderately hydrophilic (contact angle ~62° vs. ~95° bare Ti), enhance corrosion resistance in simulated body fluid, and promote carbonated apatite formation, indicative of strong osteoconductive potential [94].

This unique capability to gently process and combine diverse materials—from polymers and biomolecules to ceramics and nanoparticles—without compromising their functionality makes MAPLE exceptionally well-suited for fabricating next-generation multifunctional bioactive coatings. Preserving the activity of delicate biomolecules while embedding them within a durable matrix is paramount for achieving true clinical success in applications ranging from infection control to guided tissue regeneration. Therefore, thin films with superior properties can be fabricated using the MAPLE technique, providing effective control over surface thickness and roughness, which is particularly valuable for the development of functional coatings on medical implants or devices where enhanced surface characteristics are key for improving osteointegration, biocompatibility, and their overall performance.

#### 2.4.3. Other Laser-Enabled Strategies

Beyond PLD and MAPLE, laser techniques such as laser-induced forward transfer (LIFT) [95], laser-induced periodic surface structures (LIPSS) [96], two-photon polymerization (TPP) [97], or laser ablation [98]. LIFT enables direct patterning of biomolecules, cells, or microparticles with micrometer-scale precision, opening routes to spatially resolved biointerfaces and tissue-engineering scaffolds [73]. In particular, LIPSS provides a flexible surface modification approach enabling the formation of stable, periodic subwavelength nanostructures with minimal impact on the material, and these patterned surfaces have been shown to influence cell behavior, improve wettability, and enhance surface functionality relevant for biomedical implants and tissue-engineered interfaces [70,96]. TPP enables the fabrication of 3D microstructures from polymer composites while maintaining cytocompatibility and precise control over feature size for biomedical microdevices [99]. Laser ablation is an alternative technique to generate micro/nanostructures or coatings from bulk materials, and it is integrated with other PVD or CVD processes in hybrid lines to combine high-energy, localized modification with high-rate conventional deposition [70,100]. Large-area commercial PLD systems and cross-beam PLD geometries are being developed to improve throughput and enable metastable phases not accessible by equilibrium methods, relevant for hard, wear-resistant, and bioactive coatings [73].

In Table 1, we made a comparative assessment between laser vs. non-laser deposition techniques in order to explicitly identify similarities and differences across deposition routes. This comparison serves as a guide for selecting the appropriate technique based on the desired material class and clinical function, a theme that will be explored in detail in the following section.

## 3. Functional Thin-Film Coatings: Material Classes, Deposition Strategies and Clinical Applications

To understand the evolution of modern medical implants, one must look at the surface. While the bulk material provides the necessary strength and stiffness, thin-film or surface coatings—often only nanometers to micrometers thick—govern corrosion, ion release, protein adsorption, cell adhesion, and biofilm formation, and therefore largely dictate how the body “perceives” and integrates the device [4,119,120]. The clinical performance of an implant is therefore a direct consequence of the surface engineered upon it. By strategically choosing between metallic, ceramic, and polymeric coatings (or their hybrids), researchers can transform otherwise inert hardware into multifunctional, “smart” systems capable of resisting infection, modulating inflammation, releasing drugs, and accelerating bone healing [4,119,121].

Below is a breakdown of these material classes, their deposition or processing techniques, and their clinical roles.

### 3.1. Metallic Thin-Film Coatings

Metallic coatings represent a critical class of surface modification, especially for medical implants and devices, where mechanical durability, corrosion resistance, and long-term structural stability are key factors [122]. Comparing with polymeric or ceramic layers, metallic thin-film coatings are significantly more likely to match the mechanical properties of structural substrates, enhancing surface chemistry and performance [123]. Metallic coatings are used to compensate for the weak points of structural metals, such as stainless steel or titanium alloys—namely, insufficient corrosion resistance, ion release, and lack of intrinsic antibacterial activity [124]. Properly engineered metallic films can reduce leaching of toxic ions (e.g., Ni, Cr from stainless steels), increase hardness and wear resistance, and introduce antimicrobial functions through controlled release of ions such as Ag or Cu [125,126].

The processing of metallic coatings is dominated by PVD techniques, particularly magnetron sputtering, due to their ability to create dense, adherent films with controlled composition from a wide range of metallic and alloy targets. For instance, Ti-based coatings on stainless steel are typically produced by physical vapor deposition techniques—most commonly DC or RF magnetron sputtering and related PVD routes, such as TiN/TiO_2_/Ti-alloy coatings on 316L stainless steel applied by sputtering to improve hardness, wear, and corrosion resistance for biomedical use [127], and by other low-temperature methods such as cold-sprayed porous Ti overlays on 316L stainless steel that enhance corrosion resistance and osteogenic response [128]. In these systems, Ti-rich surface layers function as corrosion-resistant barriers that limit Ni and Cr ion release while improving wear resistance and bioactivity, thereby extending the lifetime and safety of cost-effective steel implants [127,128,129]. Such coated constructs are particularly attractive for orthopedic fixation devices and dental structural components, where stainless steel is retained for mechanical strength, but its surface biocompatibility is upgraded by a Ti-based (or Ti-family) coating [129]. Moreover, TiN/Ti, ZrN/Ti, and ZrC/Ti coatings can be deposited on titanium by PLD, leveraging PLD’s strength in stoichiometric transfer, exhibiting tailored corrosion resistance in simulated body fluids, with bare Ti and with ZrN/Ti determining a strong polarization resistance [130]. Also, pure Ti coating deposited on stainless steel exhibits high adhesion to the substrate with high reported strength. In this way, the Ti layer enables the formation of TiO_2_ nanotubes characterized by improved coating adhesion and mechanical properties due to thermal treatment, where Ti diffuses at the coating–substrate interface [131]. It is reported that variations in porosity have a significant effect on the corrosion resistance of TiO_2_ coatings on surgical steel surfaces, determining a better performance than bare steel, and enhanced corrosion resistance can be achieved by optimizing porosity and improving bonding through higher sintering temperatures [132].

Beyond simple metals and alloys, PVD’s combinatorial capabilities are used to design advanced metallic films. Metallic glasses based on Cu–Nb–Ti represent a second class of advanced metallic thin films for biomedical use. These coatings are commonly fabricated by combinatorial DC magnetron sputtering to form Cu–Nb–Ti thin-film metallic glasses (TFMGs) on additively manufactured Ti-6Al-4V implants [133]. While these TFMG coatings exhibit excellent hardness and corrosion resistance, a critical consideration for clinical translation is the potential for long-term interfacial failure if the adhesion between the amorphous coating and the crystalline substrate is not perfectly optimized. Furthermore, the long-term fatigue behavior of such dissimilar material interfaces under cyclic physiological loading remains an under-explored area requiring further investigation. Owing to their amorphous structure, these TFMGs exhibit high hardness and excellent corrosion resistance; when further combined with a bioactive glass such as S53P4 deposited as a top layer by electrophoretic deposition, the resulting architecture provides a dual-action surface that is mechanically durable and strongly bone-bonding, with enhanced apatite formation and osteoblast proliferation compared with uncoated Ti-6Al-4V [133]. Such hybrid coatings are suited for high-load orthopedic components, including hip and knee stems and other large joint implants, where simultaneous mechanical robustness and improved osseointegration are required [133,134]. Complementary to metallic glasses, metallic multilayer coatings processed by laser, using PLD’s layer-by-layer control, exhibit significant properties for biomedical interfaces. Ti/Cu/Ti and Ti/Zr/Ti thin-film multilayers determine well-defined surface morphologies promoting favorable cell responses without including cytotoxic effects, demonstrating the capability of lasers to develop surfaces that enhance the biological performances of metallic coatings for medical implants or devices [135].

**Figure 2:**
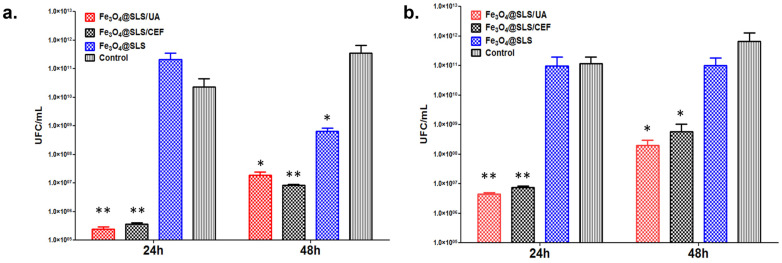
Evaluation of (**a**) *S. aureus* and of (**b**) *P. aeruginosa* biofilm growth after 24/48 h of incubation with Fe_3_O_4_@SLS, Fe_3_O_4_@SLS/CEF, and Fe_3_O_4_@SLS/UA coatings vs. control (one-way ANOVA, when comparing samples vs. control * *p* < 0.05; ** *p* < 0.001) [137].

Laser-based techniques like MAPLE are also employed to create functional metallic composite coatings, particularly where bio-organic components are involved. Fe_3_O_4_-based functionalized coatings represent a novel type of metallic coating that can improve the surface of a biomaterial [136,137]. They present a significant antimicrobial activity, acting as effective enzyme mimetics, generating free radicals that kill bacteria [137]. Functionalizing Fe_3_O_4_ nanoparticles with usnic acid (UA) and ceftriaxone (CEF), stabilizing them with sodium lauryl sulfate (SLS), and depositing them using the MAPLE technique could develop an antimicrobial thin film toxic to bacteria, enhancing the biocompatibility of medical devices. In Figure 2, the activity of the nano-modified surface to stop the development of bacterial biofilms against Gram-positive (*S. aurus)* and Gram-negative *(P. aeruginosa*) pathogens is presented. The decrease in bacterial populations compared to the uncoated surface is evident, demonstrating the key effect of the surface on the overall performance of a medical device. These properties highlight the potential of the developed magnetic nanopowder and MAPLE-deposited coatings as promising surfaces for future strategies in the field of managing chronic and recurrent biofilm-associated infections caused by these pathogenic bacteria [137]. In addition, Fe_3_O_4_ functionalized with *Nigella sativa* and dicloxacillin, synthesized by MAPLE, are highly promising candidates for enhanced wound dressings. This type of coating suggests strong antimicrobial and antibiofilm activity throughout a high drug release, followed by a sustained inhibition of film formation while remaining suitable for the support of dermal fibroblast viability due to its non-cytotoxicity and biocompatibility [138].

Finally, PVD techniques like magnetron sputtering are the workhorse for producing alloyed antibacterial metallic coatings. Antibacterial TiCu(Ag)-type films form a third important example of metallic thin-film systems. These coatings are typically deposited by magnetron sputtering from Ti–Cu–Ag or related alloy targets to generate amorphous or nanocrystalline films on metallic substrates [28,29]. The films are engineered to release low levels of Cu and/or Ag ions, which exert strong bactericidal effects against pathogens such as *Staphylococcus aureus* and *Pseudomonas aeruginosa* through membrane damage and reactive-oxygen-species generation, while maintaining acceptable fibroblast biocompatibility [55,139,140]. As a consequence, TiCu(Ag)-type coatings are promising for trauma and external fixation hardware, as well as for titanium implants more generally, in clinical scenarios where reduction in implant-associated infection is critical [55].

Overall, metallic coatings are characterized by a wide variety of materials, including pure metals, metal oxides, alloys, nitrides, metallic glasses, and multifunctional hybrid structures being synthesized by laser-based deposition methods or other surface processing techniques. Each of these types of coatings presents distinct advantages for biomedical surface engineering. In this way, their ability to integrate corrosion resistance and protection, improved biological performance, or mechanical robustness makes them highly suitable for medical implants or devices.

### 3.2. Ceramic and Bioceramic Thin Films

Ceramic thin films are widely used due to their unique properties, including high hardness, thermal stability, and chemical resistance. However, their limitations, such as inherent brittleness and low fracture toughness, restrict damage tolerance [141,142]. In order to address these limitations, systems such as ceramic-metal composites and microstructural engineering, including indium tin oxide (ITO) or zinc oxide (ZnO) doped with alumina, have been studied and developed to enhance the mechanical performance [142]. Regarding the biomedical field, ceramic thin films or bioceramic thin films exploit these characteristics while providing significant compatibility with living tissues, considering their corrosion protection and wear resistance [77]. The primary clinical function of ceramic and bioceramic thin films is to achieve robust osseointegration—the direct structural and functional connection between living bone and the surface of a load-bearing implant.

Ceramic and bioceramic thin films, particularly calcium phosphates and bioactive glasses, are central to achieving robust osseointegration—the direct structural and functional connection between living bone and the surface of a load-bearing implant. Calcium phosphate ceramics such as hydroxyapatite (HA) and bioactive glasses closely mimic the mineral phase of bone and, in physiological or simulated body fluids, form bone-like hydroxycarbonate apatite layers that provide strong, stable bonding to bone tissue [143,144,145].

To harness these materials, deposition techniques that can faithfully transfer their complex, multi-element composition are required. PLD and, to some extent, RF magnetron sputtering are the methods of choice for this task. Natural materials such as ovine and bovine HA can be deposited by PLD on titanium as novel coatings for implants [146,147]. Hydroxyapatite is widely used in forms such as powders, porous scaffolds, or granules to fill bone defects created by disease or surgical intervention, including dental and orthopedic procedures. Considering it as a temporary structural framework, it supports rapid bone ingrowth, integrates into the host tissue, and serves as an effective alternative to autologous bone grafts, thereby shortening healing time [148]. Comparing animal-origin HA deposited with thin films made from commercial HA, all deposited by PLD, it is observed that the coatings made by animal-origin HA presented a higher roughness and slightly poorer calcium composition, suggesting a faster reabsorption. In this way, these coatings are beneficial for implants due to their higher osteointegration and their low-cost approach [147].

Furthermore, the ability of PLD and sputtering to precisely control doping levels allows for the creation of enhanced, next-generation ceramic coatings. Ion-substituted hydroxyapatite is a widely studied ceramic coating within this class. PLD and RF magnetron sputtering are routinely employed to transfer complex HA and doped-HA compositions onto titanium and stainless-steel substrates with high chemical fidelity and strong adhesion [77,149]. When HA is substituted with ions such as Mg or Sr, the resulting thin films typically show enhanced osteoblast proliferation, improved in vitro bioactivity, and accelerated bone formation compared with stoichiometric HA, effects linked to modified mineralization kinetics and cell-signaling pathways [143,150,151]. Such ion-substituted HA coatings are particularly attractive for dental implants and spinal fusion cages, where rapid and robust bone–implant integration is essential [152]. Also, Co-substituted hydroxyapatite deposited on Ti-6Al04V using spin-coating exhibited improved corrosion resistance and biocompatibility. Adding Co^2+^, the system showed an improved performance overall, highlighting cell proliferation, strong adhesion, and smooth surface [153]. Nevertheless, HA and magnesium phosphate thin coatings loaded with bone morphogenetic protein (BMP4) deposited by MAPLE and then nebulized by ceftriaxone, enhanced implant osteointegration while preventing peri-implant infections, highlighting good cytocompatibility with osteoblast cells [154].

**Figure 3:**
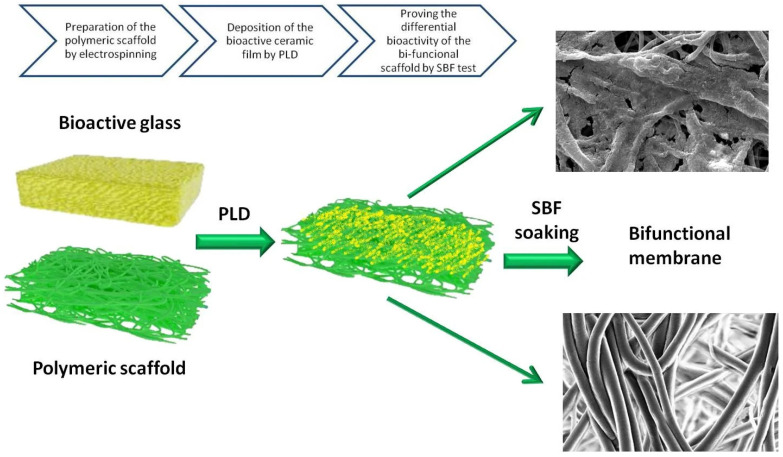
Schematic representation of the development of Mn-doped glass-ceramic bioactive thin films using combined electrospinning and pulsed laser deposition (PLD), followed by in vitro bioactivity evaluation through simulated body fluid (SBF) immersion for tissue engineering applications [157].

For creating composite coatings that combine the bioactivity of ceramics with the therapeutic action of other agents, MAPLE’s gentle processing is highly advantageous. Bioactive glass (BG) and glass-ceramic coatings constitute another important ceramic category. PLD can produce nanostructured BG and glass-ceramic films that retain complex compositions and exhibit high bioactivity, while techniques such as MAPLE and electrophoretic deposition allow fabrication of composite BG–polymer or BG–peptide coatings at relatively low temperatures [77,155]. Upon exposure to simulated body fluid, these coatings typically form a carbonated apatite layer that chemically bridges the implant surface and bone; when BG is combined with antimicrobial or bioactive agents such as melittin or vitamin D_3_ in MAPLE-deposited composites, coatings can simultaneously enhance corrosion resistance, osteoconductivity, and antibacterial performance [94,156]. Such BG-based coatings are suited for load-bearing orthopedic and dental implants and for bone scaffolds that require both strong osseointegration and improved resistance to microbial colonization [145,155]. PLD technique can be combined with electrospinning for developing Mn-doped glass-ceramic bioactive thin film for tissue engineering applications (Figure 3) [157]. PLD has the capability of precise control of composition and morphology [111] while electrospinning is a versatile tool for developing polymeric nanofibers [158]. By soaking the coated scaffolds in simulated body fluid (SBF) at a controlled temperature and pH for several periods ranging from 3, 7, 14, and up to 28 days, in vitro evaluation of the biological activity can be assessed, highlighting the formation of bone-like apatite minerals. After 28 days of immersion in SBF, the coated scaffold exhibited a uniform and densely packed layer of nanocrystalline HA covering the inner surface [157]. Thus, this combination is beneficial for developing organic-inorganic fibrous scaffolds such as poly(D, L-lactic acid) (PDLLA)/gelatin (GA) coated on the surface with a glass ceramic layer of Mn-BG exhibiting enhanced bioactivity and surface-driven biomineralization that promotes selective nucleation and growth of a dense nanocrystalline HA layer on the coated side [157].

Finally, for achieving conformal, ultra-thin ceramic coatings on complex implant geometries, Atomic Layer Deposition (ALD) is an indispensable tool. Inorganic oxide coatings such as TiO_2_ deposited by Atomic Layer Deposition (ALD) provide a third family of ceramic thin films. ALD and plasma-enhanced ALD can generate ultra-thin, highly conformal TiO_2_ layers on complex three-dimensional metallic or polymeric (e.g., PEEK or Ti-alloy) implant geometries with sub-nanometer control over thickness [159]. These conformal TiO_2_ coatings improve corrosion resistance and enable precise tuning of surface nanotopography and wettability, which enhances osteoblast adhesion and mineralized matrix formation while inhibiting adhesion and growth of multiple bacterial strains, including *Staphylococcus aureus* and *Escherichia coli* [160,161,162]. Consequently, ALD-grown TiO_2_ films are highly relevant for complex-shaped orthopedic implants and PEEK or Ti-alloy spinal inserts where conformal coverage, improved osseointegration, and antibiotic-free antibacterial behavior are desired [160,161,162].

Considering this, ceramic or bioceramic thin films are recognized as key surface-engineering solutions for modern implants because they combine mechanical protection, such as hardness, wear, and corrosion resistance, with biological performance, including bioactivity and bone bonding. Techniques such as PLD, sputtering, spin coating, MAPLE, electrophoretic deposition, and ALD enable tailored composition, morphology, thickness, and adhesion on complex substrates. Clinically, these coatings can boost osseointegration, conduct mineralization, and reduce infection risk, improving long-term orthopedic and dental implant performance.

### 3.3. Polymeric and Biopolymer Composite Films

Polymeric coatings ranging from simple barrier layers to nanotechnology-based composites are widely used in several domains, including chemical, physical, and biomedical [163,164]. Universal polymeric coatings are designed to modify a wide variety of surfaces while remaining stable under different operating conditions and independent of substrate characteristics. This versatility is achieved by relying on well-designed interactions rather than specific covalent bonds, allowing the coating to be applied to a significant range of material surfaces [163]. In the biomedical domain, polymeric coatings exhibit different characteristics that include wear resistance, improved mechanical strength, enhanced surface chemistry, biocompatibility, and improved corrosion resistance [165]. Overall, these types of coatings cover a significant number of medical applications, such as biosensors, drug delivery, cardiovascular stents, and tissue engineering [165,166]. The key clinical advantage of polymeric coatings lies in their ability to provide a “soft” and tunable interface, often serving as a reservoir for therapeutic agents. Polymeric and biopolymer composite films provide a “soft” and tunable interface between medical devices and surrounding tissues. These coatings can be readily loaded with drugs, proteins, growth factors, or nanoparticles and are often processed at low temperature by methods such as MAPLE, which preserves the chemical structure and bioactivity of sensitive polymers and biomolecules [167,168]. Other deposition techniques widely used for polymer and biopolymer films include dip coating, spin coating, and spray coating, which are cost-effective routes to homogeneous thin films, as well as Chemical Vapor Deposition (CVD) of functional polymers that yield conformal, solvent-free coatings for biomedical applications [3,110].

For creating antimicrobial surfaces, MAPLE’s gentle and solvent-free nature is ideal for co-depositing delicate organic agents with polymers. Antimicrobial polymer composites form a major subclass of these systems. Using MAPLE, metal and metal-oxide nanoparticles such as Ag or ZnO, as well as antibiotics or phytochemicals, can be embedded in polymer matrices to produce uniform composite films on polymers or metals while maintaining polymer integrity [168,169]. The development of an environmentally friendly method of biocompatible antibacterial coating has been reported, consisting of Ag NPs combined with polyethylene glycol/poly(lactide-co-glycolide), PEG/PLGA blends deposited in a simple way by the MAPLE technique. Thus, these coatings provided antibacterial activity against *E. coli,* reducing bacterial adhesion and proliferation while maintaining compatibility with the underlying substrate [170]. Moreover, the MAPLE technique enables the fabrication of high-quality silk fibroin—Poly(3-hydroxybutyric-acid-co-3-hydroxyvaleric-acid) (SF:PHBV) composite coatings with precise control of microstructure and preserved chemical structure, tailoring the degradation rate, surface wettability, and degradation rate of it only by adjusting the SF:PHBV ratio [171]. Also, the degradation behavior of polycaprolactone-polyethylene glycol blends (PCL-blend-PEG) fabricated for titanium implants using the dip-coating method has been studied. These blends exhibit a degradation behavior dependent on composition in simulated body fluid, with increasing PEG content leading to more uniform surface erosion and laminar morphologies, while higher PCL fractions promote the formation of larger pits and cavities [172]. More broadly, polymer/metal-nanoparticle composites—especially those incorporating silver or copper nanoparticles—act as local reservoirs that provide sustained release of antimicrobial ions, reducing biofilm formation while retaining overall biocompatibility [173,174]. Such antimicrobial polymer coatings are attractive for catheters, wound dressings, and high-touch medical or implant surfaces where long-term control of infection and biofilm formation is required [173,175].

**Figure 4:**
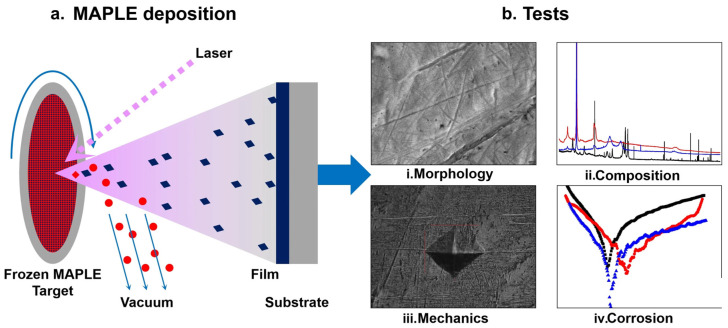
(**a**) Schematic representation of MAPLE-deposited coating of chitosan/bioglass on TiZrTaAg alloy. (**b**) Evaluation of morphology, composition, mechanical properties, and corrosion behavior of these coatings using (**i**) SEM, (**ii**) Raman spectroscopy, (**iii**) nanoindentation, and (**iv**) Tafel plots [180].

Similarly, for creating biomimetic, pro-healing surfaces, MAPLE is a key tool for depositing sensitive biopolymers like collagen and chitosan. Collagen, chitosan, and other protein-based films constitute a second important type of polymeric coating. MAPLE from frozen aqueous targets enables deposition of protein-containing and biopolymer thin films while preserving their chemical structure and functionality, making it suitable for delicate biomolecules [167,168]. Collagen-rich films mimic essential features of the extracellular matrix, spreading, and migration, and thus improve the integration of otherwise inert substrates with surrounding tissues [176,177]. Such protein-based and ECM-mimetic coatings are particularly relevant for periodontal and soft-tissue engineering scaffolds, soft tissue–implant interfaces, and functionalized metal or ceramic surfaces designed to promote tissue ingrowth [176,178]. Chitosan is also widely studied as a coating for bone-related applications due to its biocompatibility, antimicrobial activity, and functional versatility, while phosphorylated chitosan further enhances bioactivity by binding calcium ions and promoting biomimetic calcium phosphate deposition for improved remineralization [179]. Using the MAPLE technique, two types of bioactive coatings have been developed on TiZrTaAg substrates consisting of chitosan and bioglass with the addition of ZnO and graphene (Figure 4) [180]. Detailed characterization of these bioactive coatings proved the uniform morphology of deposition on the TiZrTaAg alloy using MAPLE, preserving the chemical functionality of each component, and forming thin films with a thickness of tens of nanometers and enhanced surface hardness. Thus, the structural, morphological, and corrosion results of these characterizations enhanced the suitability of chitosan and bioglass coating for medical implants and scaffold applications [180].

Finally, for creating functional, conductive coatings for biosensing, MAPLE enables the combination of polymers with advanced nanomaterials. Hybrid graphenic–polymer films exemplify a third category of polymeric and biopolymer composite coatings. MAPLE has been used to co-deposit graphene-like materials with polymers such as polydopamine or poly(vinylpyrrolidone) onto flexible substrates like PDMS, forming uniform nanometric films [168,169]. These hybrids combine the electrical conductivity and chemical robustness of graphenic materials with the adhesion, flexibility, and cytocompatibility of polymers, yielding surfaces that support fibroblast and epithelial cell viability and are suitable for biosensing and electrical stimulation contexts [168,169,181]. Such flexible, conductive graphenic–polymer composites are therefore promising for wearable, skin-contact sensors and for flexible or miniaturized neural and dental interfaces, where both mechanical compliance and electrical functionality are essential [169,182].

Polymeric coatings and bio-coatings represent highly versatile surface-engineering solutions for biomedical devices, providing tunable, enhanced, and multifunctional interfaces that complement the mechanical properties of underlying substrates. Deposition techniques such as MAPLE, dip-coating, PLD, or spin-coating enable precise control over compositions, thickness, degradation behavior, and bioactivity while protecting the performance of sensitive polymers, proteins, and composite systems. Regarding biomedical usage, such coatings support tissue integrations, enable controlled drug release, and find their application in several directions of the medical field, such as implants, biosensors, scaffolds, and wound dressing.

## 4. Challenges and Future Directions

Despite significant progress in laser-based thin film deposition for biomedical applications, several cross-cutting issues still limit clinical translation. Standardization and reproducibility remain major challenges. Variations in laser fluence, target morphology, background pressure, and substrate condition across different laboratories lead to significant variability in film properties, making results difficult to compare and complicating process qualification for regulatory acceptance. This lack of standardization remains a major bottleneck for industrial translation. Clinical translation requires compliance with ISO 10993, which provides the required characteristics and biological evaluation of medical devices, including laser-coated implants [183]. These aspects are critical for the success of implantable systems or devices. However, these aspects are rarely addressed in the initial stages of coating studies, highlighting a visible gap between regulatory validation and material development for clinical implementation. Variations in laser fluence, plume dynamics, target composition, background pressure, and target–substrate distance strongly influence growth rate, composition, morphology, and crystallinity, making results difficult to compare across laboratories and complicating process qualification and regulatory acceptance [72,184,185].

Long-term in vivo performance of laser-processed bioactive coatings is still insufficiently documented. Most reports in the literature emphasize in vitro cytocompatibility, bioactivity in simulated body fluid, and short-term antibacterial assays. However, systematic animal and clinical data on coating durability, degradation behavior, wear debris generation, ion release kinetics, and immune responses over extended implantation periods are remarkably scarce, particularly for multifunctional and nanoparticle-containing systems. Although in vitro evaluation of coatings deposited by laser techniques validates their overall biocompatibility and their capacity to modulate cell behavior, comprehensive in vivo assessment and studies are required to determine their long-term stability and performance under certain conditions [111]. Most reports emphasize in vitro cytocompatibility, bioactivity in simulated body fluid, and short-term antibacterial assays, whereas systematic animal and clinical data on coating durability, degradation behavior, wear, ion release, and immune responses are scarce, particularly for multifunctional and nanoparticle-containing systems [186]. For example, studies from the last two decades related to calcium phosphate (CaP)-based coatings deposited by PLD showed promising in vitro results, but it lacks clinical validation due to insufficient standardization of coating properties and experimental designs [187].

Scalability and cost also limit industrial uptake. PLD and MAPLE typically offer excellent stoichiometry control and versatility but are constrained by small deposition areas, micrometer-scale particulates, and modest growth rates, while more established techniques such as PVD sputtering, CVD, sol–gel, and electrophoretic deposition are easier to scale, yet struggle to integrate complex bioactive or biomolecule-rich architectures at high throughput [53,111].

Multifunctionality introduces further trade-offs. Designing coatings that simultaneously provide hardness or wear resistance, corrosion protection, strong osseointegration, antimicrobial activity, and controlled drug release often leads to competing requirements in composition, microstructure, thickness, and processing temperature that can compromise one function while optimizing another [156,186]. Also, the versatility and the significant number of laser processing parameters that must be taken into consideration for developing biomedical coatings pose a challenge in terms of reproducibility. Variations in laser fluence, ambient conditions, background pressure, and process dynamics are hard to precisely control and can significantly affect the characteristics of the coating, including the thickness of the film or microstructure [188].

Emerging directions to tackle these bottlenecks include AI-assisted optimization of deposition parameters, real-time monitoring of film growth (e.g., optical emission, in situ diffraction, or reflectometry), and hybrid processing lines that combine techniques such as sol–gel plus PVD or ALD/MLD plus MAPLE to build hierarchical, multi-material architectures [189]. In parallel, AI-driven materials design is being rapidly explored for hydrogels and soft biomaterials, suggesting similar opportunities for bioactive coatings and smart, bioresponsive interfaces that adapt to pH, enzymatic activity, or inflammatory signals [190,191].

Within this landscape, MAPLE is expected to see increasing use in high-value, personalized implants and biointerfaces. Its unique ability to gently transfer complex biomolecules, drugs, and hybrid organic–inorganic systems with preserved functionality, albeit at relatively low throughput, makes it particularly suited to patient-specific devices where precise spatial placement and integrity of bioactive agents outweigh purely economic constraints [87,111,192]. Table 2 summarizes the main actual challenges of laser-based coating technologies alongside emerging strategies proposed to overcome these limitations or barriers, highlighting the significant role of the approach used (PLD, MAPLE, or hybrid approaches).

## 5. Conclusions

In this review, we summarized the recent progress of biocompatible thin films, highlighting their importance on the surface of medical implants or devices with a particular focus on laser-based deposition approaches. We provide a comprehensive overview of current deposition techniques for biomedical applications, discussing their fundamental principles as well as their advantages and limitations, examining the key classes of thin film coatings for medical implants with particular emphasis on their specific properties that enhance the performance of medical devices, and summarizing the recent developments reported in the literature. Additionally, we discussed their current challenges and future perspectives related to scalability, reproducibility, multifunctionality, and clinical translation of biocompatible coatings deposited by laser techniques.

Thin film deposition techniques provide versatile methods to improve surface properties such as corrosion resistance, hardness, biocompatibility, and functionality, enabling the decoupling of bulk mechanical performance from surface biological behavior across a wide range of applications. Overall, thin film deposition techniques can be broadly classified into PVD, CVD, and solution-based approaches. Each of these methods has unique mechanisms, process conditions, and levels of control that need to be taken into account to complete the requirements of specific biomedical applications. PVD techniques enable the fabrication of dense, adherent, and compositionally controlled coatings with enhanced corrosion resistance, hardness, and antibacterial performance, making them particularly suitable for the biomedical domain. CVD and related methods offer high-purity, conformal, and compositionally controlled coatings on complex geometries, supporting improved mechanical performance, biocompatibility, and functionality of biomedical implants and devices. Solution-based techniques such as sol–gel, dip coating, and electrophoretic deposition remain attractive due to their low cost and scalability, enabling uniform and bioactive coatings, although these techniques have disadvantages related to thickness control, porosity, and post-deposition processing.

Laser-based deposition methods represent alternative, versatile techniques that can perform deposition of thin films with precise control. Among these techniques, the most used and versatile techniques for biomedical thin films are PLD and MAPLE. Biocompatible coatings with enhanced performance can be achieved using PLD. Coatings fabricated using this technique provide precise control over composition and microstructure, enabling the fabrication of stable, multifunctional, and biologically active surfaces that support improved long-term performance of medical implants. Using MAPLE thin films with superior and unique properties can be fabricated, providing effective control over surface thickness and roughness, which is particularly valuable for the development of functional coatings on medical implants or devices.

Functional thin-film coatings, including metallic, ceramic, polymeric, and hybrid systems, enable the precise tailoring of surface properties such as corrosion resistance, mechanical durability, biocompatibility, and biological response, making them essential tools for advanced biomedical surface engineering. Metallic coatings such as pure metals, metal oxides, alloys, nitrides, metallic glasses, and multifunctional hybrid structures deposited by laser techniques present distinct advantages for biomedical surface engineering due to their ability to integrate corrosion resistance and protection, improved biological performance, or mechanical robustness. Ceramic or bioceramic thin films are key surface-engineering solutions for modern implants, combining mechanical protection such as hardness, wear, and corrosion resistance with biological performance, including bioactivity and bone bonding. This specific class of coatings can boost osseointegration, conduct mineralization, and reduce infection risk, improving long-term orthopedic and dental implant performance. Polymeric coatings and bio-coatings represent highly versatile surface-engineering solutions for biomedical devices, providing tunable, enhanced, and multifunctional interfaces. In the biomedical field, such coatings support cell adhesion and tissue integration, enable controlled drug release, and find their application in several directions of the medical field, such as implants, biosensors, scaffolds, and tissue engineering.

In summary, this review has underscored that the selection of a deposition technique is a fundamental design decision that dictates the clinical outcome of a medical device. The central theme is that each deposition method functions as a strategic tool, offering unique capabilities to process specific material classes and thereby achieve targeted biological and mechanical performances. For example, the high stoichiometric fidelity of PLD makes it the technique of choice for depositing bioactive ceramic coatings like ion-substituted hydroxyapatite, directly leading to enhanced osseointegration and long-term implant stability. Conversely, the gentle, low-temperature nature of MAPLE is indispensable for creating multifunctional polymeric coatings that can co-deliver delicate biomolecules, providing a direct route to infection control and guided tissue regeneration without compromising the activity of therapeutic agents. Meanwhile, the high-energy, line-of-sight nature of sputtering (PVD) is strategically used to deposit hard, wear-resistant metallic coatings that ensure the mechanical longevity of load-bearing implants and can be alloyed to provide corrosion protection and antimicrobial effects.

Despite the clear advantages of laser deposition technologies for biomedical surface engineering, their broader clinical adoption remains constrained by challenges related to reproducibility, scalability, long-term performance, and the integration of multiple functionalities within a single coating system. Issues related to laser processing parameters, lack of clinical data, or sensitive requirements that have to be achieved by biomedical coatings could potentially limit the large-scale implementation and clinical translation of laser-deposited thin films. Furthermore, the critical gap between promising in vitro results and validated long-term in vivo performance must be bridged. Specific hurdles include the lack of standardized protocols for process qualification, the difficulty of scaling laboratory techniques to industrial throughput, and the critical gap between promising in vitro results and validated long-term in vivo performance. Furthermore, issues such as sterilization compatibility, regulatory approval pathways (e.g., ISO 10993 compliance), and the mechanical fatigue behavior of coated devices under physiological loading remain insufficiently addressed. Nevertheless, there are some promising routes to overcome these limitations, such as real-time monitoring of deposition parameters, enhanced design of multifunctional coating architectures, or personalized implants. Continued progress is expected from the combination of advanced process monitoring, data-driven optimization, and hybrid deposition strategies, which could enable more reliable, scalable, and application-specific laser-deposited coatings for novel medical implants and devices. Ultimately, by framing these deposition techniques not as mere fabrication steps but as the primary design tools for engineering clinical success, the field can move towards more rational and effective development of next-generation biomedical devices.

## Figures and Tables

**Table 1:** Comparative analysis: laser vs. non-laser techniques.

Key Parameter	Laser-Based (PLD/MAPLE)	PVD (Sputtering/Arc/PED)	CVD/ALD/MLD	Sol–Gel/EPD/Dip-Coating	Ref.
Stoichiometry and Material Complexity	Excellent for complex oxides, glasses; MAPLE preserves organics and biomolecules	Very good for metals/alloys and many ceramics; flexible co-sputtering	Excellent atomic-scale control; ideal for conformal inorganic and hybrid films	Good for oxides, glasses; sensitive to precursor chemistry and processing	[41,50,83,85]
Biomolecule/Drug Incorporation	PLD limited by heat; MAPLE highly suitable for proteins, antibiotics, peptides, and natural compounds	Possible via low-energy sputtering or multilayers; risk of denaturation	Challenging at high T; ALD/MLD enable some organic–inorganic hybrids	Good for some polymers and drugs; stability and burst release are concerns	[33,73,81,84]
Film Density, Adhesion, and Roughness	Dense, well-adhered films; nanoscale roughness tunable via fluence and gas; MAPLE may yield more porous morphologies MAPLE (as surface pretreatment for adhesive joints): Relative ↑ in joint strength; absolute MPa not specified [101]	High density and adhesion, especially for hard coatings; good stress control PVD (sputtering, evaporation, etc.): ~50–180 MPa [102] PVD—bioceramics on metals: ≥15–25 MPa (pull-off) [103]	Dense and conformal; excellent coverage of complex topologies CVD/PE-CVD (SiOx, PEO-like, etc.): Similar order to PVD; often limited by substrate, not film [104,105]	Often porous; adhesion improved by multilayers or post-treatments Sol–gel (F-HA on Ti-6Al-4V): ~20–30 MPa [106,107]	[19,35,62,74]
Processing Temperature	Moderate; can coat polymers and temperature-sensitive substrates; local heating near plume (RT: (20–25 °C)–400 °C)	Generally low–moderate; compatible with many metals and some polymers (RT—800 °C)	Wide range; often high for thermal CVD, lower for PECVD/ALD/MLD (300–1000 °C)	Low to moderate; may require calcination or sintering steps (RT—600 °C)	[39,52,67,86,108,109]
Scalability and Cost	PLD: Lab-to pilot-scale; small coating areas and high cost limit mass production MAPLE: Currently, small areas, relatively slow and costly, mainly research and niche device applications	Industrial vacuum tools; widely scalable for hard coatings, but with higher equipment cost and lower rate than some solution methods	Mature in industry for some sectors (e.g., semiconductors); expensive, complex; scaling mainly limited by reactor size but based on well-understood heat/mass transport	Sol–gel: High throughput and low start-up cost; considered commercially attractive for large-area coatings Dip-coating: Simple, low-cost, easily scaled to large or 3D parts and stents	[25,39,48,60]
Thickness control	PLD: ~0.05–5 µm; dense, often stoichiometric layers; thickness tuned by pulse number and fluence MAPLE: Thin films with good thickness control via pulse number and rate; nanometers–hundreds of nm	Nanometer–micrometer, good control via power, time, and pressure; Å to µm films routinely achieved	Precise, down to <20 nm; excellent conformality on complex 3D and porous substrates	Sol–gel (solution-based): Typically <1 µm per layer; thickness increased by multilayer deposition and calcination Dip-coating: Thickness set by solution viscosity and withdrawal speed; micrometer to sub-micrometer films	[41,50,110,111]
Tunability of composition/structure	PLD: High: laser fluence, background gas, and target choice enable control of crystallinity, morphology, and doping MAPLE: Very high for soft/organic systems: preserves functional groups, controls roughness, and allows multilayers and gradients (combinatorial MAPLE)	High: Adjust target, power, pressure, and reactive gas to tune phase, stress, morphology, and properties	Very high: Monomer/precursor choice, temperature, and pressure allow tailoring of chemistry, crosslinking, wettability, permeability, and responsiveness	Sol–gel: Moderate–high: composition, porosity, and roughness tuned by sol chemistry and thermal treatment Dip-coating: Moderate: solution composition readily varied; multilayers possible, but fine nanoscale compositional control limited vs. vapor methods	[35,37,50,110,112,113]
Deposition rate	0.5 Å/s–0.1 nm/s (PLD) 0.1 nm/s–1 nm/s (MAPLE)	0.1 nm/s–100 nm/s	1 nm/s–100 nm/s (CVD) 0.1 nm/s–1 nm/s (ALD, MLD)	1 nm/s–10 nm/s	[73,86,108,114,115,116,117]
Typical biomedical functions/applications	PLD: Stoichiometric inorganic bioactive coatings (e.g., hydroxyapatite, bioglass) on metallic implants for accelerated bone integration; silver- or other ion-doped antibacterial films; nanoparticle-based antibacterial and wound-healing coatings MAPLE: Gentle deposition of polymers, biomolecules, drugs, and hybrid organic–inorganic coatings while preserving bioactivity; drug-eluting films (e.g., rapamycin), biocompatible graphenic and polymer coatings on flexible devices and catheters, biomimetic and gradient coatings for osseointegration, antibacterial protection, and biosensors	Hard, wear- and corrosion-resistant coatings on orthopedic and dental implants; improved osseointegration; reduced wear debris; antibacterial/antifouling and biocompatible surfaces on surgical, cardiovascular, and neurosurgical devices	Conformal functional coatings to enhance wear resistance and friction, improve biocompatibility and hemocompatibility; antifouling/antimicrobial surfaces; barrier and separation membranes; surfaces for biosensing, drug delivery, and tissue engineering scaffolds	Sol–gel: Bioactive and porous calcium-phosphate or bioglass-type layers on implants to promote osseointegration and bone regeneration; low-temperature drug delivery and biofunctional coatings Dip-coating: Simple application of polymer or hybrid layers for drug-eluting stents and devices, antibacterial and bioactive surface layers, and other liquid-processed functional films on medical components	[37,41,50,53,84,111,118]

**Table 2:** Current challenges of laser-based thin-film deposition for biomedical applications.

Challenge	Issue	Impact on Clinical Translation	Emerging Solutions	Ref.
Standardization and reproducibility	Sensitivity in laser fluence, target composition, and background pressure	Poor laboratory reproducibility	In situ diagnostics, developing an AI assistant parameter	[72,184,185,193]
Limited vivo performance	Lack of clinical data; studies are focused more on in vitro tests	Uncertain durability or degradation	Long-term animal studies, standardized in vivo protocols	[41,186,194]
Scalability and cost	Small deposition areas, low growth rates	Limited industrial adoption	Developing large-area PLD or cross-beam PLD	[53,72,111,195]
Multifunctionality trade-offs	Several conflicting requirements (e.g., bioactivity, drug release, and hardness)	Performance and functionality compromise	Developing hybrid approaches, multilayer architectures	[156,186,196]
Production of biomolecule-loaded coatings	Gentle depositions (usually by MAPLE)	Restricted personalized devices	Personalized implants	[190,191,197]
Regulatory and clinical translation	Sterilization compatibility; ISO 10993 biocompatibility; fatigue performance under physiological loading; lack of standardized in vivo protocols	Delays in approval, risk of mechanical failure, and non-compliance with safety standards	Early alignment with ISO/ASTM/ISO 10993; combined mechanical–biological testing in simulated body environments; development of consensus in vivo testing protocols	[37,103,105,198,199]

## Data Availability

No new data were created or analyzed in this study. Data sharing is not applicable to this article.
